# The Impact of Physical Activity on Food Reward: Review and Conceptual Synthesis of Evidence from Observational, Acute, and Chronic Exercise Training Studies

**DOI:** 10.1007/s13679-020-00372-3

**Published:** 2020-04-15

**Authors:** Kristine Beaulieu, Pauline Oustric, Graham Finlayson

**Affiliations:** Appetite Control & Energy Balance Research Group, School of Psychology, Faculty of Medicine & Health, University of Leeds, Leeds, West Yorkshire UK

**Keywords:** Physical activity, Exercise, Food reward, Appetite, Liking and wanting, Obesity management

## Abstract

**Purpose of Review:**

This review brings together current evidence from observational, acute, and chronic exercise training studies to inform public debate on the impact of physical activity and exercise on food reward.

**Recent Findings:**

Low levels of physical activity are associated with higher liking and wanting for high-energy food. Acute bouts of exercise tend to reduce behavioral indices of reward for high-energy food in inactive individuals. A dissociation in liking (increase) and wanting (decrease) may occur during chronic exercise training associated with loss of body fat. Habitual moderate-to-vigorous physical activity is associated with lower liking and wanting for high-fat food, and higher liking for low-fat food.

**Summary:**

Food reward does not counteract the benefit of increasing physical activity levels for obesity management. Exercise training appears to be accompanied by positive changes in food preferences in line with an overall improvement in appetite control.

## Introduction

Among the reasons that people with obesity cite for avoiding exercise is a lack of enjoyment and perceived failure to lose weight [1, 2]. Moreover, there is a misconception that persists among some individuals that exercise is counter-productive for weight management. This common assertion is reinforced by occasional reports in the media about exercise and food reward [3, 4]. Biological explanations, reliant on soft evidence, have been put forward suggesting that glycogen depletion, reduced blood glucose levels, endorphin release or other signals generated during exercise can increase appetite or cause specific cravings for foods. Alternatively, psychological accounts propose that high fat or sugary foods are sought out post-exercise to counteract negative affect or reward virtuous behavior. Research over the past 10 years has shown that physical activity and eating behavior are loosely coupled, but the physiological and neurocognitive mechanisms that contribute to this relationship are complex [5]. Moreover, the evidence for the impact of physical activity on food reward is difficult to assess due to the absence of randomized controlled trials and differences between study designs—encompassing observational, acute, and chronic interventions. Differences also exist in the modality and intensity of physical activity examined and the variety of methodologies used to measure reward responses to food. In a review of longitudinal weight management interventions that measured food reward outcomes at baseline and follow-up, Oustric and colleagues [6••] identified 17 studies consisting of dietary, pharmacological, cognitive, and exercise-based intervention types. Overall, a post-intervention reduction in food reward was found across all treatment types—except exercise, where no consistent changes were reported. While it is interesting to speculate that exercise training may have a moderating influence on the relationship between weight management and food reward, only three exercise studies were eligible for inclusion in this systematic review. Moreover, interpretation was limited by small sample sizes, lack of a control condition in one study, and inconsistent use of food reward measures. Further research is needed to update and summarize the available evidence from observational, acute, and chronic study designs to gain an overview of the influence of physical activity and exercise on food reward. With this objective in mind, the specific aims of this review were to describe the role of physical activity and food reward in appetite control and weight management; to tabulate and synthesize the different types of evidence that have addressed the impact of physical activity on food reward; and to discuss, and where possible harmonize, the findings in the light of relevant moderators and methodological issues. To our knowledge, there is currently no comprehensive review of the literature on physical activity and food reward. A critical examination of the evidence may help to clarify some of the perceived barriers for engaging in physical activity and exercise for obesity management.

## Current Thinking on the Role of Physical Activity in Appetite Control

In the last 5 years, evidence has accrued showing that physical activity affects both episodic (meal-to-meal) and tonic (basal) homeostatic mechanisms that influence appetite control [7]. Acute exercise influences gastric emptying [8], and attenuates the release of ghrelin and increases the secretion of satiety peptides, e.g., peptide YY, glucagon-like peptide-1, and pancreatic polypeptide [9]. Chronic exercise improves body composition [10–12], and leptin and insulin sensitivity [13–15].

Our research has shown that habitual physical activity has a small positive association with daily energy intake (accounting for around ~ 3% of the variance) [16], but this is only logical when considering the increase in energy requirements and the longer-term indirect effects from increased resting metabolic rate after changes in fat-free mass. It has been proposed that chronic exercise influences appetite control through an increase in hunger but also a strengthening of post-meal satiety [17]. Indeed, exercise and physical activity appear to interact with nutritional factors to enhance satiety signaling, with studies showing that people who engaged in more exercise and physical activity were better able to compensate for differences in the energy content of food (achieved by increasing the ratio of fat to carbohydrate) at a subsequent meal than their less active counterparts [18]. While more active individuals are driven to eat more due to their greater energy requirements, their stronger satiety response to food appears to allow for a better matching between energy intake and energy expenditure [19••]. This relationship between physical activity level and daily energy intake is best represented by a J-shaped curve, whereby individuals with low levels of physical activity on the left of the curve have dysregulated appetite with greater intake than expenditure, and individuals with greater levels of physical activity towards the right of the curve have a proportional increase in intake with increasing expenditure. These findings suggest that concerns about exercise or increased levels of physical activity being counter-productive for obesity management should be concerned with non-homeostatic and food reward-related mechanisms that may be driving unhealthy food choices.

## Defining Food Reward and Its Importance for Weight Management

Food reward is important for understanding appetite control and has the logical status of an intervening variable that guides eating behavior. Food reward is encoded by distinct neural pathways in the brain and can be modulated by metabolic signaling, sensory stimuli from the food environment and cognitive processes such as attention, learning, and memory [20]. Food reward is often conceptualized to consist of two distinct sub-components—“liking” and “wanting.” These have been broadly studied [21], following extensive work demonstrating their dissociation in the brain and behavior of many species including humans [22]. Liking is the sensory pleasure exerted while eating a food and wanting is rather the, often implicit, drive to eat triggered by a food cue [23]. Liking and implicit wanting for energy-dense foods are related to excess energy intake in free-living and laboratory settings [23, 24]. However, liking accounts only for a small proportion of the variance in food intake [25, 26], and unconscious processes such as implicit wanting may play a larger role in driving overeating [27, 28]. Food reward is an important factor in weight management through its intervening status between the nutritional requirements of the body and stimulation from the food environment [23], but it is likely that liking and implicit wanting sometimes act separately to influence appetite control [6••].

While several techniques to measure food reward have been developed [21] (e.g., reinforcing value tasks, willingness to pay), the Leeds Food Preference Questionnaire (LFPQ) has been designed to measure both liking and implicit wanting for distinct dimensions (e.g., fat and sweet taste) of foods common in the diet. Food reward measured by the LFPQ can be interpreted as both a state- and a trait-dependent measure depending on the timing and condition of measurement. Indeed, liking and wanting have been shown to be partially dissociable pre to post food consumption according to sensory-specific satiety [29]. On the contrary, food cravings, defined here as spontaneous instances of strong explicit wanting for a specific food, tend to be measured as a trait reflecting the frequency, intensity or quality of cravings experienced over a specified time period [30–32]. Neural activation to foods measured through functional magnetic resonance imaging (fMRI) are also used as an inference of food reward and this technique allows the analysis of different regions of interest and their functional connectivity [33]. However, fMRI measures of reward should be used in conjunction with behavioral measures or actual food intake to support their interpretation.

## Review of Literature

### Methods

The inclusion and exclusion criteria for this non-systematic review were established prospectively. We included studies with a general, healthy population of adults (≥ 18 years) or children (< 18 years), including those with overweight or obesity. Studies that included adults or children with overweight or obesity who were specifically trying to lose weight were also included. We excluded studies in populations with diseases (including substance related and addictive disorders), in vitro and animal studies. The interventions and comparisons of interest included any type of structured exercise or comparisons between different physical activity levels. All chronic training studies had to have a minimum intervention duration of 4 weeks. The outcomes of interest included all psychometric measures of food reward obtained either directly (e.g., ratings or pleasantness or desire to eat) or indirectly (e.g., measure of the willingness to work to obtain a food, reaction time), as well as neuronal response to food cues measured by fMRI. We included unpublished and ongoing studies where relevant.

The search strategy for this review combined electronic searches and hand searching. To identify ongoing or completed, but unpublished trials, ClinicalTrials.gov was searched on 22 November 2019 and researchers known to be active in the topic were contacted. Limits were set to include all papers published in English or French after 2009, in healthy human samples. Authors were contacted for additional information if unclear or not reported in the manuscript.

### Observational Studies of Physical Activity and Exercise

As shown in Table 1, only three studies have examined food reward differences in defined active and inactive groups [34, 35, 38]. Horner et al. [38] found that in the fed state, overall liking, and liking for high-fat savory, high-fat sweet, and low-fat sweet food measured by the LFPQ was lower in active compared with inactive men differing in BMI, and differences for foods overall and low-fat sweet foods remained after adjusting for differences in percentage body fat. In both fed and hungry states, active men had a greater implicit wanting for low-fat savory foods compared with inactive men, but only the differences in the fed state remained after adjusting for percentage body fat. Faster gastric emptying was found to be associated with greater liking for savory food and lower implicit wanting for high-fat food. Two studies in men and women differing in physical activity levels but matched for BMI (~ 23 kg/m^2^) found no differences in liking or wanting for high-fat relative to low-fat food in the hungry or fed states [34, 35].

**Table 1:** Description of the observational, acute, and chronic training studies examining the impact of physical activity on food reward

Reference	Participant characteristics	Exercise/physical activity details	Food reward method	Food reward results	Associations with appetite outcomes
Observational studies					
Beaulieu et al. 2017 [34] UK	Sex: females and males BMI status: < 29.9 kg/m^2^ Active: *n* = 20 (50% males) Age: 30 (10) years BMI: 22.6 (1.9) kg/m^2^ Inactive: *n* = 19 (42% males) Age: 30 (9) years BMI: 23.1 (2.7) kg/m^2^	PA level assessment**:** IPAQ and PA monitor (SenseWear) Active: ≥ 4 exercise sessions/week (MVPA = 182 (67) min/day) Inactive: ≤ 1 exercise session/week (MVPA = 103 (37) min/day) Exercise session: ≥ 40 min MVPA	- LFPQ: liking and implicit wanting bias for fat - Setting: laboratory - State: pre and post high-fat or high-carbohydrate lunch (ad libitum)	- No difference in food reward (liking and wanting) between groups. - Difference between liking/wanting: none	- Food intake: energy intake greater in high-fat relative to high-carbohydrate in both groups. - Eating behavior traits: tendency for restraint to be higher in HiPA compared with LoPA - Body composition: HiPA had lower body fat percentage than LoPA
Beaulieu et al. 2017 [35] UK	Sex: females and males BMI status: < 29.9 kg/m^2^ HiMVPA: *n* = 12 (33% males) Age: 29 (10) years BMI: 22.4 (2.1) kg/m^2^ ModMVPA: *n* = 11 (27% males) Age: 26 (3) years BMI: 22.7 (2.2) kg/m^2^ LoMVPA: *n* = 11 (27% males) Age: 30 (11) years BMI: 23.1 (2.9) kg/m^2^	PA level assessment: Tertiles of daily MVPA measured by PA monitor (SenseWear) HiMVPA: 174 (39) min/day ModMVPA: 121 (15) min/day LoMVPA: 83 (16) min/day	- LFPQ: liking and implicit wanting bias for fat - Setting: laboratory - State: pre and post preloads—HE (~ 700 kcal) and LE (~ 260 kcal) relative to water control (0 kcal)	- No difference in food reward (liking and wanting) between groups - Difference between liking/wanting: none	- Food intake: greater reduction in liking and wanting after HE preload relative to LE preload in all groups. ModMVPA and HiMVPA reduced ad libitum energy intake at the lunch meal following consumption of the HE compared with the LE preload, while the LoMVPA group did not. No effect of MVPA group on energy intake at dinner or evening snack box. - Eating behavior traits: no differences between groups - Body composition: no differences between groups
Drummen et al. 2019 [36•] Netherlands	Sex: females and males BMI status: > 25 kg/m^2^ *n* = 39 (56% males) Age: 53 (11) years BMI: 32.3 (3.7) kg/m^2^ Participants had impaired fasting glucose and/or glucose tolerance	PA level assessment: Baecke questionnaire (work, sport, and leisure-time physical activity) [37] Work = 2.6 (0.8) range 1.3–4.3 Sport = 2.6 (0.7) range 1.0–4.0 Leisure = 2.9 (0.7) range 1.5–4.4 Scores ranging from 1 (low level of PA) and 5 (high level of PA)	- fMRI: BOLD signals to high-energy food, low-energy food and non-food images - Setting: laboratory - State: after overnight fast	- Inverse association between leisure-time PA and food compared with non-food brain activation in the right thalamus, left middle cingulate gyrus, right precuneus, left putamen, and left angular gyrus. - Positive association between work-related physical activity and brain activation (disappeared after adjusting for BMI and age) - No association with sport-related physical activity. - Difference between liking/wanting: NR	- Food intake: NR - Eating behavior traits: positive association between brain activation and disinhibition. No association with restraint or susceptibility to hunger. - Body composition: no association
Horner et al. 2016 [38] Australia	Sex: males BMI status: 18–40 kg/m^2^ Active: *n* = 22 Age: 29 (8) years BMI: 24.5 (2.6) kg/m^2^ Inactive: *n* = 22 Age: 31 (9) years BMI: 27.4 (4.2) kg/m^2^	PA level assessment: Self-report and PA monitor (ActiGraph) Active: ≥ 4 exercise sessions/week (PA = 709 (239) kcal/day) Inactive: ≤ 1 exercise session/week (PA = 525 (185) kcal/day) Exercise session: ≥ 40 min MVPA	- LFPQ: liking and implicit wanting for HFSW, LFSW, HFSA, LFSA - Setting: laboratory - State: post fixed breakfast (400 kcal) and pre lunch 5 h later	- Fed: Active had lower liking for high-fat foods, low-fat sweet foods and for foods overall compared with inactive. Active had greater wanting for low-fat savory foods. - Hungry: No differences in liking between active and inactive. Active had greater wanting for low-fat savory foods. - Fed to hungry: Active had greater increase in liking for all food categories combined than inactive. - Difference between liking/wanting: Faster gastric emptying associated with liking for savory foods and slower gastric emptying associated with greater implicit wanting for high-fat foods.	- Gastric emptying: Inverse association with post-prandial changes in liking low-fat savory foods. Positive association with liking taste bias in hungry state (i.e., faster gastric emptying associated with greater liking for savory foods). No association with liking fat bias nor implicit wanting taste bias. Positive association with implicit wanting fat bias in fed and hungry states. Effects independent of body fat.
Killgore et al. 2013 [39] USA	Sex: females and males BMI status: 19.8–34.8 kg/m^2^ *n* = 37 (59% males) Age: 30 (8) years BMI: 24.5 (3.7) kg/m^2^	PA level assessment: Self-reported habitual PA in min/week (typical days/week × duration/day) PA = 151 (160) min/week (range 0–540)	- fMRI: response to high-energy food, low-energy food and non-food images - Subjective food preferences: desire to eat depicted food item at that moment (VAS) - Setting: laboratory - State: 1 h fasted (pre scan food intake 323 (245) kcal)	- Inverse association between habitual PA and fMRI responses (medial orbitofrontal cortex and left insula) to high-energy foods relative to low-energy foods - Inverse association between habitual PA and preference for high-energy savory foods	- Subjective food preference: fMRI responses positively associated with preference for high-energy savory foods (not for high-energy sweet foods). No association between fMRI responses to high-energy relative to low-energy foods and preference for low-energy foods.
Luo et al. 2018 [40•] USA	Sex: females and males BMI status: NR *n* = 40 (48% males) Lean individuals: *n* = 22 (46% males) Age: 21 (2) years BMI: 22.6 (1.9) kg/m^2^ Individuals with obesity: *n* = 18 (50% males) Age: 22 (2) years BMI: 35.2 (4.0) kg/m^2^	PA level assessment: Self-reported from 3 to 5 24-h recalls over 2 months (mean daily minutes of MVPA i.e., activities ≥ 3 METs) Lean individuals: MVPA = 125 (84) min/day Individuals with obesity: MVPA = 134 (114) min/day	- fMRI: responses to high-energy food and non-food images - Setting: laboratory - State: 9–11 am after overnight fast, task performed 20–30 min after 75 g glucose ingestion	- Inverse association between MVPA and brain response to food cues in middle insula and left postcentral gyrus. - Individuals with obesity: inverse association between MVPA and brain responses. - Lean individuals: non-significant inverse association between MVPA and brain responses. - Association between MVPA and brain responses stronger in males than females.	- Food intake: NR - Eating behavior traits: NR - Body composition: NR
Oustric et al. 2018 [41] UK	Sex: females BMI status: 18.5–45.0 kg/m^2^ *n* = 156 Age: 53 (11) years BMI: 32.3 (3.7) kg/m^2^ Pooled data from 6 studies	PA level assessment: Quintiles of daily MVPA measured by PA monitor (SenseWear) Q1: 25 (8) min/day Q2: 53 (9) min/day Q3: 83 (9) min/day Q4: 120 (12) min/day Q5: 197 (62) min/day	- LFPQ: liking and implicit wanting bias for fat/taste - Setting: laboratory - State: hungry (3–10 h fast)	- MVPA inversely associated with liking and implicit wanting fat bias. - MVPA positively associated with liking taste bias. - Q5 greater liking and wanting for low-fat foods, while Q1-Q3 greater liking and wanting for high-fat foods. - Difference between liking/wanting: none	- Food intake: NR - Eating behavior traits: No association between PA and food cravings. Craving for sweet food (Control of Eating Questionnaire; CoEQ) positively associated with explicit liking and implicit wanting for sweet foods on the LFPQ. Craving for savory foods (CoEQ) associated with LFPQ explicit wanting for savory foods. Craving control (CoEQ) negatively associated with implicit wanting for high-fat foods. - Body composition: Fat mass index (FMI) but not waist circumference (WC) was inversely associated with explicit liking and implicit wanting for sweet relative to savory foods. WC was positively associated with all liking and implicit wanting for high-fat relative to low-fat foods. FMI was also associated with implicit wanting for high-fat foods.
Acute exercise studies					
Alkahtani et al. 2014 [42] Australia	Sex: males BMI status: > 25 kg/m^2^ PA level: sedentary (criteria NR) *n* = 12 Age: 29 (4) years BMI: 29.1 (2.4) kg/m^2^	- Intensity: moderate-intensity interval training (MIIT; alternating between ± 20% FAT_max_) vs. high-intensity interval training (HIIT; 85% VO_2peak_) - Type: cycle ergometer - Duration: MIIT 5-min stages at ±20% FAT_max_ for 30 min, HIIT 15-s intervals and 15-s recovery (workload matched to MIIT; ~ 18 min) - Timing: morning - Control condition: no; MIIT vs. HIIT	- LFPQ: liking and implicit wanting for HFSW, LFSW, HFSA, LFSA - Setting: laboratory - State: before and after exercise (after an overnight fast)	- Decrease in wanting and increase in liking for all the food categories independent of the intensity - Difference between liking/wanting: liking increased while wanting decreased but without a control this response might be due to the effect of time rather than exercise	- Food intake: NR - Eating behavior traits: NR - Body composition: NR
Alkahtani et al. 2019 [43] Saudi Arabia	Sex: males BMI status: NR PA level: moderately active (2–5 h structured aerobic exercise/week) *n* = 14 (8 for food reward data) Age: 24 (6) years BMI: 23.4 (3.3) kg/m^2^	- Intensity: moderate (60% VO_2max_) interspersed with low (30% VO_2max_) - Type: contraction type eccentric (downhill running at − 12% inclination) vs. concentric (flat running) - Duration: 5 stages of 8 min at 60%VO_2max_/2 min at 30%VO_2max_ - Timing: morning - Control condition: yes; no exercise	- LFPQ: liking and implicit wanting bias for fat/taste - Setting: laboratory - State: before, after exercise and 24 h after exercise (before an ad libitum lunch)	- No change in food reward after exercise - Difference between liking/wanting: greater liking of savory foods over sweet foods in downhill running than front running	- Food intake: no change - Eating behavior traits: NR - Body composition: NR
Crabtree et al. 2014 [44] UK	Sex: males BMI status: 21.8–26.6 kg/m^2^ PA level: moderately active (2 h/week) *n* = 16 Age: 23 (3) years BMI: 24.2 (2.4) kg/m^2^	- Intensity: high (70% VO_2max_) - Type: treadmill run - Duration: 60 min - Timing: morning - Control condition: yes; no exercise	- fMRI: BOLD signals to high- and low-energy food cues compared with non-food pictures - Setting: laboratory - State: fasted	- Decreased activation in the pallidum for high-energy food and increase for low-energy food after exercise - Difference between liking/wanting: NR	- Food intake: NR - Eating behavior traits: NR - Body composition: NR
Evero et al. 2012 [45] USA	Sex: females and males BMI status: < 25 kg/m^2^ PA level: habitually active (> 3 h/week) *n* = 30 (57% males) Age: 22 (4) years BMI: 23.6 (2.2) kg/m^2^	- Intensity: high (83% HR_max_) - Type: cycle ergometer - Duration: 60 min - Timing: morning - Control condition: yes; no exercise	- fMRI: BOLD signals to high- and low-energy food cues compared with neutral control - Setting: laboratory - State: fMRI was performed after exercise after an 8–12 h overnight fast	- Exercise reduced the neuronal response to food cues in brain regions related with food reward (i.e., insula, putamen, rolandic operculum) - Difference between liking/wanting: decrease in regions related to liking and wanting but no behavioral measures	- Food intake: NR - Eating behavior traits: NR - Body composition: NR
Farah et al. 2012 [46] UK	Sex: females and males BMI status: no limits PA level: NR *n* = 27 (52% males) Female: *n* = 13 Age: 26 (3) years BMI: BMI: 22.8 (3.1) kg/m^2^ Male: *n* = 14 Age: 36 (10) years BMI: 26.1 (3.3) kg/m^2^	- Intensity: moderate (6 METs) - Type: treadmill walk - Duration: 60 min - Timing: morning - Control condition: yes; no exercise	- VAS: liking - Setting: laboratory - State: immediately, 60, 120, and 180 min after exercise (overnight fasted)	- No change in liking - Difference between liking/wanting: wanting not measured	- Food intake: NR - Eating behavior traits: NR - Body composition: NR
Finlayson et al. 2009 [47] UK	Sex: females BMI status: < 25 kg/m^2^ PA level: 2.4 (1.2) engagements/week *n* = 24 Age: 24 (6) years BMI: 22.3 (2.9) kg/m^2^	- Intensity: moderate (70% HR_max_) - Type: cycle ergometer - Duration: 50 min - Timing: morning - Control condition: yes, no exercise	- LFPQ: relative preference (food choice), liking and implicit wanting for HFSW, LFSW, HFSA, LFSA - Setting: laboratory - State: before and after exercise (2 h after a fixed breakfast, kcal NR) and after an ad libitum lunch 30-min post exercise	- Increase in implicit wanting after exercise in those who compensated or ate more at the ad libitum lunch in response to exercise - Difference between liking/wanting: Changes in implicit wanting but not liking	- Food intake: After exercise some individuals increased their energy intake (compensators) and had enhanced implicit wanting - Eating behavior traits: NR - Body composition: NR
Martins et al. 2015 [48] Norway	Sex: females and males BMI status: > 25 kg/m^2^ PA level: sedentary (criteria NR) *n* = 12 (42% males) Age: 33 (10) years BMI: 32.3 (2.7) kg/m^2^	- Intensity: HIIT and ½ HIIT (all out; average ~ 85% HR_max_), continuous (70% HR_max_) - Type: HIIT vs. ½ HIIT vs. continuous cycling - Duration: HIIT (8-s intervals and 12-s recovery for 250 kcal; ~ 18 min), ½ HIIT (8-s intervals and 12-s recovery for 125 kcal; ~ 9 min), continuous exercise (250 kcal; ~ 27 min) - Timing: morning - Control condition: yes; no exercise	- LFPQ: relative preference (food choice), liking and implicit wanting bias for fat - Setting: laboratory - State: before and after exercise/ before an ad libitum lunch (standardized breakfast of 600 kcal consumed 1 h before exercise/rest)	- No change in food reward - Difference between liking/wanting: no differences	- Food intake: no change - Eating behavior traits: NR - Body composition: NR
McNeil et al. 2015 [49] Canada	Sex: females and males BMI status: < 25 kg/m^2^ PA level: inactive (<150 min/week) *n* = 16 (50% males) Age: 22 (3) years BMI: 22.8 (1.8) kg/m^2^	- Intensity: high (aerobic 70% VO_2peak_, resistance 70% 1-repetition maximum) - Type: aerobic vs. resistance - Duration: aerobic ~ 24 min, resistance ~ 86 min (matched for energy expenditure at 4 kcal/kg; ~ 275 kcal) - Timing: morning - Control condition: yes; no exercise	- LFPQ: relative preference (food choice), liking and implicit wanting bias for fat/taste - Setting: laboratory - State: pre and post ad libitum lunch 30 min after exercise (standardized breakfast of 534 kcal consumed ~ 1.5 h before exercise)	- Decrease in the relative preference for high-fat relative to low-fat foods after both exercise - Decrease in liking for high-fat foods following resistance, but not aerobic - Difference between liking/wanting: change in food choice and liking, but not implicit wanting	- Food intake: no change - Eating behavior traits: NR - Body composition: no difference in bodyweight
Miguet et al. 2018 [50] France	Sex: females and males (adolescents) BMI status: > 29.9 kg/m^2^ PA level: inactive (<2 h/week) *n* = 33 (36% males) Age: 13 (1) years BMI: 35.0 (4.3) kg/m^2^	- Intensity: high (70%, 75%, 80%, 85%, and 90% HR_max_) - Type: high-intensity interval training cycling - Duration: 5 × 2-min increasing intensity intervals followed by 30-s recovery (15 min) - Timing: morning - Control condition: yes; no exercise	- LFPQ: relative preference, liking and implicit wanting bias for fat/taste - Setting: laboratory - State: pre and post ad libitum lunch 30 min after exercise (standardized breakfast of 500 kcal consumed 2.5 h before exercise)	- Decrease in implicit wanting for sweet in the exercise condition vs. increase in the control - Difference between liking/wanting: implicit wanting but not liking is decreasing	- Food intake: decrease in energy intake at lunch and dinner - Eating behavior traits: NR - Body composition: NR
Thackray et al. unpublished UK	Sex: females and males BMI status: 18.5–29.9 kg/m^2^ PA level: habitually active *n* = 32 Age: 23 (2) years BMI: 23.9 (2.6) kg/m^2^	- Intensity: self-determined moderate-to-high intensity (RPE of 15 “hard”) - Type: swimming vs. cycling - Duration: 6 × 8-min intervals with 2-min recovery - Timing: morning - Control condition: yes, no exercise	- LFPQ: relative preference (food choice), liking and implicit wanting bias for fat/taste - Setting: laboratory - State: post-exercise (3 h after fixed breakfast of 650 kcal for males, 525 kcal for females, and before ad libitum lunch meal)	- Tendency for a main effect of trial for implicit wanting fat bias (post hoc: cycling < control, cycling < swimming). - No impact of swimming or cycling on other food reward parameters. - Difference between liking/wanting: Changes in implicit wanting but not liking	- Food intake: increase in ad libitum energy intake after swimming but not after cycling - Eating behavior traits: NR - Body composition: NR
Thivel et al. 2019 [51] France	Sex: females and males BMI status: < 25 kg/m^2^ PA level: moderately active (150–240 min/week) *n* = 19 (52% males) Age: 21 (1) years BMI: 22.3 (2.9) kg/m^2^	- Intensity: low 50% VO_2max_, high 75% VO_2max_ - Type: cycling - Duration: low intensity 45 min, high intensity 30 min - Timing: morning - Control condition: yes; no exercise	- LFPQ: relative preference, liking and implicit wanting bias for fat/taste - Setting: laboratory - State: pre and post fixed lunch 30 min after exercise (females 750 kcal and males 900 kcal; standardized breakfast of 500 kcal consumed 3 h before exercise).	- No change in food reward - Difference between liking/wanting: no differences	- Food intake: (self-reported) no change - Eating behavior traits: NR - Body composition: NR
Saanijoki et al. 2018 [52] Finland	Sex: males BMI status: 19.9–26.9 kg/m^2^ PA level: NR *n* = 24 Age: 27 (5) years BMI: 23.5 (1.6) kg/m^2^	- Intensity: moderate (74% HR_max_) - Type: aerobic cycling - Duration: 60 min - Timing: NR - Control condition: yes; no exercise	- fMRI: BOLD signals to palatable and non-palatable foods compared with neutral control (cars) - Setting: laboratory - State: post-exercise fasted for 3 h before the scans	- No effects of exercise vs rest on neuronal responses. - Individual variability in the BOLD signals after exercise might be explained by changes in the brain opioid system. Participants who showed most increases in endogenous opioid release also had highest anticipatory fMRI reward responses following the exercise - Difference between liking/wanting: not assessed	- Food intake: NR - Eating behavior traits: NR - Body composition: NR
Chronic exercise training studies					
Alkahtani et al. 2014 [53] Australia	Sex: males BMI status: ≥ 25 kg/m^2^ PA level: inactive (criteria NR) *n* = 10 Age: 29 (4) years Moderate-intensity interval training (MIIT): BMI baseline = 30.7 (3.5) kg/m^2^ BMI post = 30.8 (3.5) kg/m^2^ High-intensity interval training (HIIT): BMI baseline = 30.9 (3.2) kg/m^2^ BMI post = 30.9 (3.2) kg/m^2^	- Frequency: 3 days/week for 4 week (cross-over with 6-week washout) - Intensity: MIIT ± 20% workload at 45% VO_2peak_, HIIT 90% VO_2peak_ - Type: MIIT vs. HIIT - Duration: 30–45 min (MIIT 5-min stages alternating between ± 20% workload, HIIT 30-s intervals and 30-s recovery) - Timing: NR - Supervision: yes - Control group: no; MIIT vs. HIIT	- LFPQ: liking and implicit wanting for HFSW, LFSW, HFSA, LFSA - Setting: laboratory - State: pre and post 45-min cycling at 45% VO_2max_ - Measurement time points: week 0 and week 4 in each intervention	- Tendency for explicit liking for high-fat non-sweet foods after acute exercise to increase with MIIT and decrease with HIIT. - No changes in wanting. - Difference between liking/wanting: yes; changes in liking but not wanting	- Food intake: No effects of training on food intake or energy intake. Tendency for fat intake (g) and % energy from fat to increase after MIIT. - Eating behavior traits: NR - Body composition: NR
Beaulieu et al. 2019 [54] UK	Sex: females and males BMI status: 26.0–38.0 kg/m^2^ PA level: inactive (≤ 2 h/week) Exercisers: *n* = 46 (35% males) Age: 43 (8) years BMI baseline = 30.5 (3.8) kg/m^2^ BMI post = 29.9 (4.0) kg/m^2^ Controls: *n* = 15 (40% males) Age: 41 (11) years BMI baseline = 31.4 (3.7) kg/m^2^ BMI post = 31.8 (3.9) kg/m^2^	- Frequency: 5 days/week for 12 weeks - Intensity: 70% HR_max_ - Type: aerobic (treadmill, rower, cycle ergometer, and elliptical) - Duration: 500 kcal (males ~ 40–45 min, females ~ 60 min) - Timing: NR - Supervision: yes - Control group: yes; no exercise	- LFPQ: liking and implicit wanting bias for fat - Setting: laboratory - State: pre and post fixed lunch (high-fat or high-CHO; 800 kcal) - Measurement time points: week 0 and week 12	- Decrease in wanting after training - No change in liking - Difference between liking/wanting: yes; changes in wanting but not liking	- Food intake: reduction in high fat ad libitum dinner intake but no change in daily high-fat energy intake [55] - Eating behavior traits: decrease in disinhibition and binge eating score - Body composition: reduction in body weight and percentage body fat, but not associated with changes in wanting
Cornier et al. 2012 [56] USA	Sex: females and males BMI status: > 25 kg/m^2^ PA level: NR *n* = 12 (42% males) Age: 38 (10) years BMI baseline = 33.3 (4.3) kg/m^2^ BMI post = NR	- Frequency: 5 days/week for 24 weeks - Intensity: up to 75% VO_2max_ - Type: treadmill - Duration: up to 500 kcal/day (40–60 min/day) - Timing: NR - Supervision: yes - Control group: no	- fMRI: responses to food vs non-food cues - Setting: laboratory - State: fasted without exercise for 24 h (chronic exercise) and fasted within 30 min of acute exercise (chronic+acute; 500 kcal 60–75% VO_2max_ for 40-60 min) - Measurement time points: week 0 and week 24	- Chronic exercise: reduction in neuronal responses observed in the bilateral parietal cortices, left insula and visual cortex. - Chronic + acute exercise: intermediate attenuation of the response to visual food cues in brain region important in food regulation compared with chronic exercise and baseline - Difference between liking/wanting: NR	- Food intake: self-reported energy intake lower after training compared with baseline but no change in macronutrient intake. No association with changes in neuronal responses. - Eating behavior traits: no change in dietary restraint or disinhibition - Body composition: reduction in body fat percentage. Changes in anterior insula responses positively associated with changes in body mass and fat mass.
Finlayson et al. 2011 [57] UK	Sex: females and males BMI status: 26.0–38.0 kg/m^2^ PA level: inactive (≤2 h/week) Responders (non-compensators): *n* = 20 (43% males) Age: 41 (9) years BMI baseline = 32.3 (4.3) kg/m^2^ BMI post = 30.9 (4.3) kg/m^2^ Non-Responders (compensators): *n* = 14 (50% males) Age: 37 (12) years BMI baseline = 29.7 (2.2) kg/m^2^ BMI post = 29.3 (2.5) kg/m^2^	- Frequency: 5 days/week for 12 weeks - Intensity: 70% HR_max_ - Type: aerobic (treadmill, rower, cycle ergometer, and elliptical) - Duration: 500 kcal (males ~ 40–45 min, females ~ 60 min) - Timing: NR - Supervision: yes - Control group: no	- LFPQ: relative preference (food choice), liking, explicit wanting for HFSW, LFSW, HFSA, LFSA - Setting: laboratory - State: pre and post acute exercise (scheduled session) - Measurement time points: week 0 and week 12	- Increase in liking after exercise in non-responders compared with responders at baseline and week 12. - Increase in explicit wanting for high-fat sweet foods in non-responders. - Increase in relative preference for high-fat sweet food in non-responders. - Difference between liking/wanting: implicit wanting NR	- Food intake: NR - Eating behavior traits: NR - Body composition: greater fat mass loss in responders
Finlayson et al. unpublished UK	Sex: females and males BMI status: 26.0–38.0 kg/m^2^ PA level: inactive (≤ 2 h/week) Non-compensators: *n* = 15 (33% males) Age: 42 (8) years BMI baseline = 30.7 (4.9) kg/m^2^ BMI post = 29.1 (5.0) kg/m^2^ Compensators: *n* = 15 (33% males) Age: 41 (9) years BMI baseline = 31.8 (3.7) kg/m^2^ BMI post = 32.1 (3.9) kg/m^2^ Controls: *n* = 15 (33% males) Age: 41 (11) years BMI baseline = 31.4 (3.7) kg/m^2^ BMI post = 31.8 (3.9) kg/m^2^	- Frequency: 5 days/week for 12 weeks - Intensity: 70% HR_max_ - Type: aerobic (treadmill, rower, cycle ergometer, and elliptical) - Duration: 500 kcal (males ~ 40–45 min, females ~ 60 min) - Timing: NR - Supervision: yes - Control group: yes, no exercise	- LFPQ: liking and implicit wanting bias for fat - Setting: laboratory - State: pre and post fixed lunch (high-fat or high-CHO; 800 kcal) - Measurement time points: week 0 and week 12	- Non-compensators showed a smaller liking and implicit wanting for high-fat food. - At baseline, compensators showed a strong liking and wanting for high-fat food whereas non-compensators showed no difference between high-fat and low-fat food. - Greater baseline reward for high-fat food in compensators reduced following the exercise intervention. - In the non-compensators, small increase in liking for high-fat food after exercise training, but a simultaneous decrease in wanting for high-fat food. - Difference between liking/wanting: yes, non-compensators increased liking but decreased wanting for high-fat food post-intervention.	- Food intake: tendency for non-compensators to decrease ad libitum dinner meal size from baseline to post-intervention that was not seen in compensators. - Eating behavior traits: NR - Body composition: significant reduction in BMI, body mass, fat mass and WC in non-compensators, whereas no changes in compensators, and increase in body mass and WC.
Martin et al. 2019 [58] USA	Sex: females and males BMI status: 25–45 kg/m^2^ PA level: inactive (< 20 min < 3 days/week) 8 kcal/kg body weight/week (KKW): *n* = 59 (27% males) Age: 48 (11) years BMI baseline = 31.4 (4.6) kg/m^2^ BMI post = NR (~ 31.3 kg/m^2^) 20 KKW: *n* = 51 (29% males) Age: 49 (12) years BMI baseline = 30.6 (4.4) kg/m^2^ BMI post = NR (~ 30.0 kg/m^2^) Control: *n* = 61 (26% males) Age: 50 (1) years BMI baseline = 32.3 (4.8) kg/m^2^ BMI post = NR (~ 32.2 kg/m^2^) Pooled exercisers (*n* = 110) divided (median split) into compensators/non-compensators based on actual and predicted weight loss.	- Frequency: 3–5 days/week (self-selected) for 24 weeks - Intensity: 65–85% VO_2peak_ (self-selected) - Type: treadmill - Duration: 8 KKW ~ 35 min/session (~ 700 kcal/week) vs. 20 KKW ~ 55 min/session (~ 1760 kcal/week) - Timing: NR - Supervision: yes - Control group: yes; no exercise	- Food Preference Questionnaire [59] preferences for food classified as alongside 2 components: fat (2 factors: High Fat and Low Fat) and carbohydrate (3 factors: High Simple Sugar, High Complex CHO, and Low CHO/High Protein) - Setting: laboratory - State: NR - Measurement time points: week 0 and week 24	- 8 KKW group increased preference for high fat/high CHO foods whereas 20 KKW group decreased. - Compensators decreased preferences for high CHO, low fat, and low fat/high CHO foods whereas non-compensators increased. - Difference between liking/wanting: NR	- Food intake: adjusted doubly-labeled water energy intake increased in both exercise groups (relative to control group). No changes in test meal energy intake. - Eating behavior traits: greater reduction in disinhibition in non-compensators relative to compensators. - Body composition: difference in body mass and body fat percentage loss between 20 KKW and control.
Martins et al. 2017 [60] Norway	Sex: females and males BMI status: ≥ 30 kg/m^2^ PA level: inactive (<1 day/week MVPA, <20 min/day < 3 days/week light PA) HIIT: *n* = 16 (13 completers; 40% males) Age: 34 (8) years BMI baseline = 33.2 (3.5) kg/m^2^ BMI post = NR ½HIIT: *n* = 16 (9 completers; 80% males) Age: 34 (7) years BMI baseline = 32.4 (2.9) kg/m^2^ BMI post = NR Continuous: *n* = 14 (13 completers; 60% males) Age: 33 (10) years BMI baseline = 33.3 (2.4) kg/m^2^ BMI post = NR	- Frequency: 3 days/week for 12 weeks - Intensity: HIIT and ½ HIIT (85–90% HR_max_), continuous (70% HR_max_) - Type: HIIT vs. ½ HIIT vs. continuous cycling - Duration: HIIT (8-s intervals and 12-s recovery for 250 kcal; ~ 20 min), ½ HIIT (8-s intervals and 12-s recovery for 125 kcal; ~ 10 min), continuous exercise (250 kcal; ~ 32 min). - Timing: NR - Supervision: yes - Control group: no; HIIT vs. ½ HIIT vs. continuous	- LFPQ: relative preference (food choice), liking and implicit wanting bias for fat/taste - Setting: laboratory - State: pre and post fixed breakfast (600 kcal) - Measurement time points: week 0 and week 12	- No effect of exercise on liking or wanting - Difference between liking/wanting: no	- Food intake: no change [61] - Eating behavior traits: NR - Body composition: reduction in body weight and trunk and leg % fat mass [61]
Miguet et al. under review France	Sex: females and males (adolescents) BMI status: ≥ 95th percentile for sex and age PA level: inactive (<2 h/week) *n* = 30 (23% males) Age: 13 (1) years BMI baseline = 35.7 (4.5) kg/m^2^ BMI post = 30.9 (5.0) kg/m^2^	- Frequency: 4 days/week for 10 months - Intensity: NR - Type: various (aerobic, strength, aquatic and leisure-time activities) - Duration: 60 min - Timing: NR - Supervision: yes - Control group: no As part of an inpatient multidisciplinary weight loss program	- LFPQ: liking and implicit wanting for HFSW, LFSW, HFSA, LFSA - Setting: laboratory - State: pre and post lunch (ad libitum) - Measurement time points: baseline, 5 months, 10 months	- Hungry: Increase in liking at 5 months followed by decrease at 10 months (similar to baseline values). No change in wanting. - Fed: Decrease in liking at 5 and 10 months. No change in wanting. - Hungry to fed: Decrease in liking at 5 and 10 months, but not baseline. No change in wanting. - Difference between liking/wanting: yes; decrease in liking, no change in wanting	- Food intake: decrease in lunch EI at 10 months - Eating behavior traits: decrease in uncontrolled eating and emotional eating at 5 and 10 months - Body composition: decrease in percentage fat mass at 5 and 10 months
Riou et al. 2019 [62] Canada	Sex: females (premenopausal) BMI status: > 27 kg/m^2^ PA level: inactive (< 150 min/week) Low intensity: *n* = 11 week Moderate intensity: *n* = 10 Age: 31 (11) years BMI baseline = 35.1 (6.2) kg/m^2^ BMI post = NR (~ 35.5 kg/m^2^)	- Frequency: 5days/week for 12–14 weeks - Intensity: LOW (40% VO_2reserve_) vs. moderate (MOD; 60% VO_2reserve_) - Type: aerobic (treadmill or cycle ergometer) - Duration: to 300 kcal (LOW ~ 62 min, MOD ~ 46 min) - Timing: NR - Supervision: 3/5 days - Control group: no; LOW vs. MOD	- LFPQ: liking and implicit wanting bias for fat/taste - Setting: laboratory - State: post breakfast/pre exercise (ad libitum breakfast on first session and quantities replicated on subsequent sessions; LOW ~ 648 kcal, MOD ~ 746 kcal) and post rest (week 4) or exercise (scheduled session; weeks 1 and 12–14) - Measurement time points: week 4, week 1, and week 12–14 (in line with menstrual cycle)	- Decrease in wanting for fat after training. - Increase in liking for savory foods pre to post-exercise at week 1 but decrease at week 12–14. - Difference between liking/wanting: yes; changes in wanting but not liking for fat with training	- Food intake: no change - Eating behavior traits: increase in susceptibility to hunger - Body composition: group by time interaction for body weight and fat mass showing increase in MOD and decrease in LOW
Thivel et al. 2019 [63] France	Sex: females and males (adolescents) BMI status: > 90th percentile for sex and age PA level: NR Eccentric: *n* = 12 (50% males) Age: 14 (1) years BMI baseline = 34.8 (5.5) kg/m^2^ BMI post = 29.0 (4.5) kg/m^2^ Concentric: *n* = 12 (50% males) Age: 13 (1) years BMI baseline = 31.8 (3.8) kg/m^2^ BMI post = 27.6 (4.0) kg/m^2^	- Frequency: 3 days/week for 12 weeks (+2 h/week physical education) - Intensity: 50% up to 70% VO_2peak_ - Type: eccentric vs. concentric cycling - Duration: 30 up to 45 min - Timing: NR - Supervision: yes - Control group: no; eccentric vs. concentric Phase 2 (week 12–24) of a 24-week inpatient multidisciplinary weight loss program	- LFPQ: relative preference (food choice), liking and implicit wanting bias for fat/sweet - Setting: laboratory - State: fasted - Measurement time points: baseline, week 12 and week 24	- Eccentric group: increase in preference for high-fat foods and savory foods (decrease in sweet bias). Increase in implicit wanting for savory foods (decrease in sweet bias). - Group by time interaction showed concentric group increased preference and implicit wanting for sweet foods while eccentric decreased. - Difference between liking/wanting: yes; changes in implicit wanting for sweet foods, no change in liking.	- Food intake: total daily energy intake increased in both groups from baseline to week 24, but only increased in concentric group from week 12 to 24. - Eating behavior traits: NR - Body composition: greater percentage body fat loss after eccentric vs. concentric exercise from week 12 to 24

Data are means (SD). *CoEQ*, Control of Eating Questionnaire; *FMI*, fat mass index; *fMRI*, functional magnetic resonance imaging; *LFPQ*, Leeds Food Preference Questionnaire; *LFSA*, low-fat savory; *LFSW*, low-fat sweet; *HFSA*, high-fat savory; *HFSW*, high-fat sweet; *HIIT*, high-intensity interval training; *MET*, metabolic equivalent of a task; *MVPA*, moderate-to-vigorous physical activity; *NR*, not reported; *PA*, physical activity; *VAS*, visual analogue scales; *WC*, waist circumference

In terms of correlational studies, objectively measured MVPA was found to be inversely associated with liking (*r* = − 0.25, *p* < 0.001) and wanting (*r* = − 0.27, *p* = 0.001) for high-fat relative to low-fat food measured by the LFPQ in 156 women across a range of BMI [41]. This is in line with a study showing that self-reported physical activity was associated with reduced fMRI responses to high-energy relative to low-energy food [39]. Another fMRI study in participants ranging in BMI found an inverse association between self-reported moderate-to-vigorous physical activity and brain response to food cues [40•]. This relationship appeared to be more prominent in the participants with obesity than the lean participants. In individuals with overweight/obesity and impaired fasting glucose and/or glucose tolerance, food compared with non-food brain activation was negatively associated with leisure-time physical activity [36•]. Interestingly, there was a positive association between brain activation and work-related physical activity, which lost statistical significance after adjusting for BMI and age, and there was no association with sport-related physical activity.

Overall, these studies suggest a reduction in food reward (both liking and wanting) with increasing levels of habitual physical activity, particularly at higher levels of adiposity. Those who accumulate more time in moderate-to-vigorous physical activity tend to prefer low-fat food and those who engage in more sedentary activity prefer high-fat food. These findings do not seem to be restricted to those who perform structured exercise. The inter-relationships between food reward, physical activity, and adiposity remain to be fully disentangled, as well as the mechanisms underlying these observed effects. One simple explanation could be that avoidance of high-fat food perceived to be unhealthy, preference for low-fat food and greater physical activity levels are all driven by a dispositional desire to be healthy and engage in a range of positive health behaviors [64, 65]. Alternatively, it has been proposed that physical activity may act as a reward “buffer” against liking and wanting for high-fat foods, whereas low levels of physical activity may render people more susceptible to hedonic eating [66, 67]. The possibility of stronger inverse associations between physical activity and food reward in lean groups versus groups with obesity suggests that exercise and leisure-time physical activity may be an effective strategy for controlling hedonic eating in people with obesity.

### Acute Exercise Studies

At least eight studies have investigated the effect of acute exercise on food reward measured by the LFPQ which allows direct comparison of outcomes (Table 1). All these studies had a no-exercise control group except for Alkahtani et al. [42], but the exercise varied in intensity (low to high), modality (aerobic vs resistance; cycling vs swimming) or in muscle contraction type (eccentric vs concentric). Two studies showed a decrease in food reward after acute exercise compared with the sedentary control. McNeil and colleagues revealed a decrease in the preference for high-fat relative to low-fat food independently of the modality of exercise (aerobic or resistance) in inactive adults within the normal range of BMI [49]. Miguet and colleagues showed a decrease in implicit wanting for sweet relative to savory food in response to an ad libitum meal after a session of high intensity interval exercise in inactive adolescents with obesity [50]. Interestingly, no change in food intake was observed in the McNeil study whereas a decrease in total energy intake (both lunch and dinner) was noted in the Miguet study. This might be related to the fact that changes in implicit wanting are a greater driver of food intake than changes in liking. Also, the McNeil study might have been underpowered to detect a change in implicit wanting. Four studies showed no changes in food reward (fat or taste bias for liking, relative preference or implicit wanting). One study compared eccentric vs concentric exercise on moderately active men (BMI 23.4 ± 3.3 kg/m^2^) and showed no effect on either appetite sensations, appetite-related hormones, or food reward [43]. Three studies compared the intensity of exercise (low versus high [51] or high- or moderate-intensity intermittent cycling [42, 48]). Two reported no effects on food reward or food intake [48, 51] whereas one found a decrease in wanting and increase in liking after both high- and moderate-intensity exercise [42]. Of note, these studies were conducted in moderately active normal-weight adults [51] and inactive adults with overweight/obesity [42, 48]. A recently completed study compared bouts of swimming or cycling to a no-exercise control in a within-subjects design (Thackray et al. unpublished). While they found that energy intake was increased after swimming but not cycling compared with control, no differences in food reward were detected except for a tendency for a main effect of trial for implicit wanting fat bias with wanting being smaller after cycling relative to swimming and control. Lastly, Finlayson and colleagues demonstrated that implicit wanting was increased in response to moderate-intensity exercise only in those individuals that increased their energy intake relative to no exercise (i.e., compensators) [47]. Consequently, even with the same methodology to assess food reward, the response to acute exercise seems to be equivocal and subject to individual variability. This could be explained by methodological issues; even though the same tool is used, the studies were conducted in different countries (UK, Saudi Arabia, Canada, France, and Norway) and the food images used may not have been cross-culturally validated [68]. The sample sizes were relatively small for most of the studies (ranging from 12 to 33), including mainly both genders and with different ranges of BMI. However, it can be noticed that exercise seems to affect food reward more clearly in inactive individuals compared with active ones in both adolescents and adults. One tentative hypothesis could be that inactive individuals (within the non-regulated zone of the J-shape curve) would benefit more from acute exercise than active individuals for whom the appetite control system is more sensitive.

Farah and colleagues used a computer-based task to measure the effect of acute exercise on liking (visual analogue scale) and other non-homeostatic indicators (ideal portion size, food utility, hunger) [46]. They found that a 60-min moderate-intensity exercise bout reduced hunger and ideal portion size but not liking. This is in concordance with previous results showing that implicit wanting rather than liking might be influenced by exercise. However, the physical activity level of the participants was not reported, and implicit wanting was not measured.

Acute exercise has also been shown to have an effect on brain reward measured by BOLD response to food cues with fMRI. Evero and colleagues showed that a 60-min high-intensity exercise bout decreased the neuronal response to food cues in brain regions related to food reward, visual attention, and inhibitory control [45]. Interestingly, regions related to both liking (i.e., insula, orbitofrontal cortex) and wanting (i.e., putamen) were reduced after exercise. This is in line with another study that found that exercise increases neural responses in reward-related regions in response to images of low-calorie foods and suppresses activation during the viewing of high-calorie foods [44]. These central responses were associated with exercise-induced changes in peripheral signals related to appetite control and hydration status. However, liking and implicit wanting were not measured directly in these studies (i.e., behavioral measures) nor was food intake. Lastly, Saanikoji et al. found no effect of an acute moderate-intensity aerobic exercise on brain food reward in lean men [52]. However, they showed that individuals who increased the most in endogenous opioid release had the highest brain reward response after the exercise compared with the control. Consequently, the opioid system might contribute to explain some individual variability in the food reward responses to exercise.

### Chronic Exercise Training Studies

As mentioned above, our recent systematic review on weight management interventions found limited evidence on the impact of exercise interventions on food reward [6••]. Among the included studies, two investigated the impact of high-intensity interval exercise compared with either moderate-intensity interval training [53] or continuous training [60]. Using a cross-over design, Alkahtani et al. [53] found that in response to acute exercise after a 4-week exercise intervention, liking for high-fat savory food seemed to increase after MIIT and decrease after HIIT in men with overweight or obesity (interaction *p* = 0.09). Another study in individuals with obesity found no changes in food reward in response to a breakfast meal (hungry and fed states) after a 12-week intervention of either high-intensity interval exercise (125 or 250 kcal, 3 days/week) or moderate-intensity continuous exercise (250 kcal, 3 days/week) [60]. In another study, no changes in liking or implicit wanting for high-fat relative to low-fat food in the hungry state were observed after a 12-week aerobic exercise intervention (500 kcal, 5 days/week), although a trend towards a reduction in wanting was noted [69]. However, more recent analyses with a non-exercising control group revealed that in response to a high-fat and high-carbohydrate fixed lunch (hungry and fed states), overall implicit wanting decreased after the 12-week exercise intervention, whereas no changes in explicit liking were found [54]. This is corroborated by another study that found a decrease in implicit wanting for high-fat relative to low-fat food in women who underwent a 3-month exercise intervention (300 kcal, 5 days/week) [62]. When food reward was measured in response to an exercise bout post-intervention in that study, there was also a decrease in liking for savory foods whereas liking for savory foods increase after acute exercise at baseline. Thus, it appears that chronic exercise training may modulate the food reward response to acute exercise in inactive individuals, also shown in an fMRI study [56].

Interestingly, changes in food reward in the study by Beaulieu et al. [54] were not associated with changes in body weight or composition, suggesting a potential independent effect of exercise. Indeed, an inverse association was found between changes in leptin (adjusted for percentage body fat) and changes in liking, but not wanting, for high-fat food [69]. Thus, leptin may have a role in mediating changes in food reward during exercise training in individuals with overweight/obesity. In contrast, a 6-month exercise intervention led to a reduction in the fasted neuronal response to food compared with non-food, and some of these changes were associated with changes in fat mass, body weight and leptin [56]. These findings are interesting in light of the seminal study by Rosenbaum [70], who reported that leptin replacement after > 10% weight loss using a liquid formula diet, modulated food cue-elicited neuronal activation in reward-related regions (consistent with wanting), but did not affect liking for the diet. Beyond leptin, it is known that weight loss also impacts fasting levels of ghrelin. For example, the RESOLVE study (NCT00917917) showed that long-term physical activity may reverse the early enhancing effect of body weight loss on plasma ghrelin [71]. Future studies should examine whether favorable effects of exercise-induced weight loss on food reward can be partly explained by modulation of ghrelin as well as leptin.

**Fig. 1:**
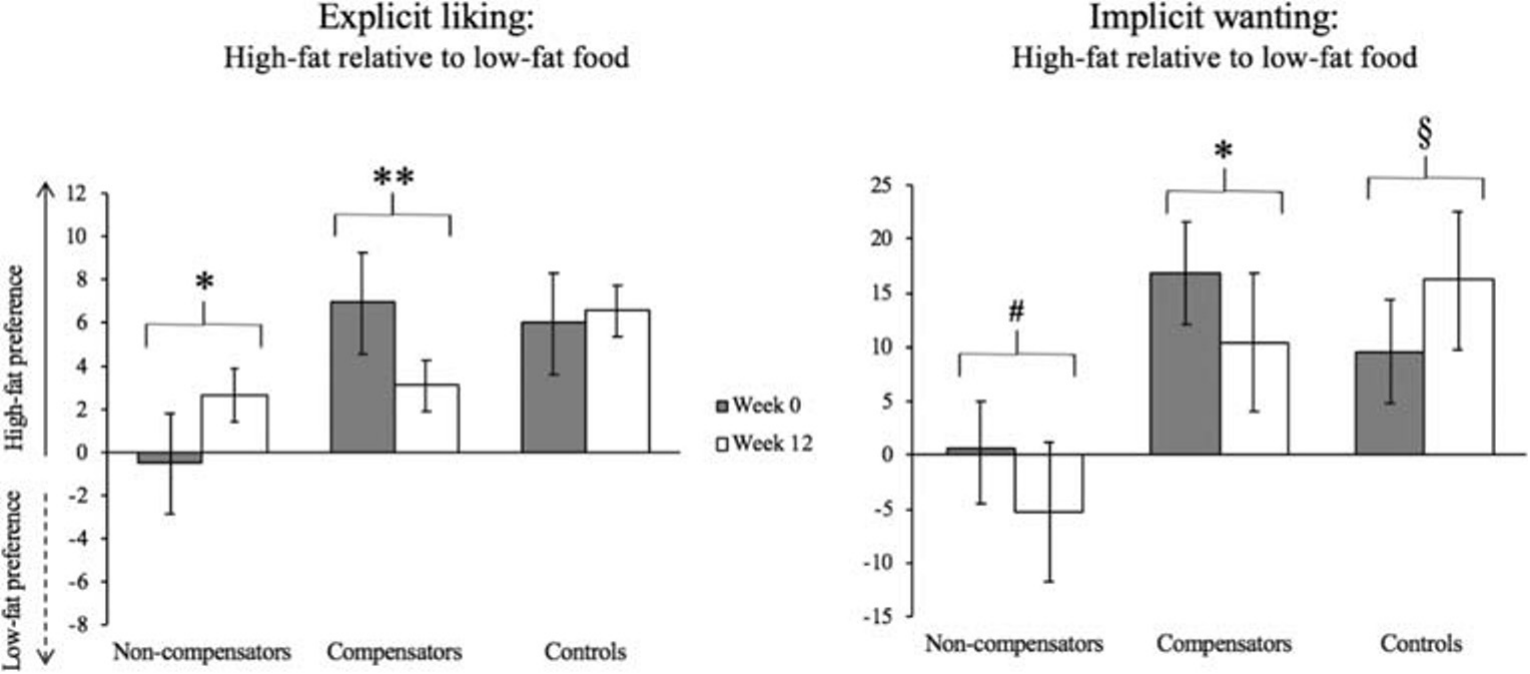
The impact of a 12-week aerobic exercise intervention (70% HR_max_, 500 kcal/day, 5 days/week) on liking and implicit wanting for high-fat food in weight loss non-compensators (*n* = 15), compensators (*n* = 15), and non-exercising controls (*n* = 15) (Finlayson et al. unpublished). *p < 0.05, **p < 0.01, ^#^*p* = 0.08, ^§^*p* = 0.10

Differences in food preferences have also been found between individuals who compensated (less than expected weight loss based on median split) compared with non-compensators during a 6-month intervention expending either ~ 700 kcal/week or ~ 1760 kcal/week [58]. Compensators had reduced preference for low-fat and high-carbohydrate food relative to non-compensators. Indeed, the hedonic response to acute exercise also appears to impact weight loss outcomes during chronic exercise training [57]. We have shown that after acute exercise, liking for all foods and wanting for high-fat sweet food increased only in compensators to a 12-week exercise intervention (those who achieved less than expected weight loss), compared with no change in non-compensators (those achieving at or above expected weight loss). These differences were independent of the exercise intervention and weight loss [57] and add to other evidence of improved appetite control in this group [72]. In a further unpublished study from our laboratory where sub-groups of 12-week exercise intervention non-compensators and compensators were compared with a non-exercising control group (Fig. 1), we observed that prior to the exercise intervention, compensators showed a strong liking and wanting for high-fat food whereas non-compensators showed no difference between high-fat and low-fat food. Secondly, we found that the greater reward for high-fat food in compensators reduced following the exercise intervention, compared with no change in controls. Lastly, there appeared to be a unique pattern of change in liking and wanting in the non-compensators who showed a small increase in liking for high-fat food after exercise training, but a simultaneous decrease in wanting for high-fat food.

In adolescents with obesity, eccentric cycling exercise as part of a 12-week inpatient multidisciplinary weight loss intervention increased the relative preference for high-fat food and increased both the relative preference and implicit wanting for savory food, whereas no changes were observed in response to concentric exercise training [63]. Another study in adolescents with obesity showed that during a 10-month inpatient multidisciplinary weight loss intervention including physical activity, liking for food in the hungry state increased from baseline to 5 months, then returned to baseline values at 10 months, whereas liking for food in the fed state decreased (Miguet et al., under review). There were no changes in wanting observed.

These studies are suggestive that chronic exercise improves food reward (reduced response to high-energy foods and increased response to low-energy foods). However, the effect sizes were relatively small and inter-individual variability tended to be large. Two studies found a reduction in implicit wanting for high-fat relative to low-fat foods after exercise training [54, 62]. This may be a result of a direct effect of exercise on brain regions related to food reward, as shown by the fMRI studies included in the current review, and others [73, 74]. Furthermore, as exercise affects cognition and executive function, it has been proposed that processes such as inhibitory control could have a moderating effect on wanting and modulate eating behavior [66].

Another two studies found an increase in liking after exercise training, which might be explained by concomitant improvements in homeostatic appetite control in these studies (a small increase in hunger or a reduction in fasting leptin concentrations). Individual differences in food reward appear to act as pre-existing moderators of the impact of exercise training on weight loss and suggest that those with healthier preferences or better satiety signaling at baseline appear to lose more weight with exercise. No clear evidence exists regarding the optimal mode, frequency, intensity, duration, and time of day for exercise to have the most impact on food reward. Further systematic research into these factors is warranted.

## Conclusions

**Fig. 2:**
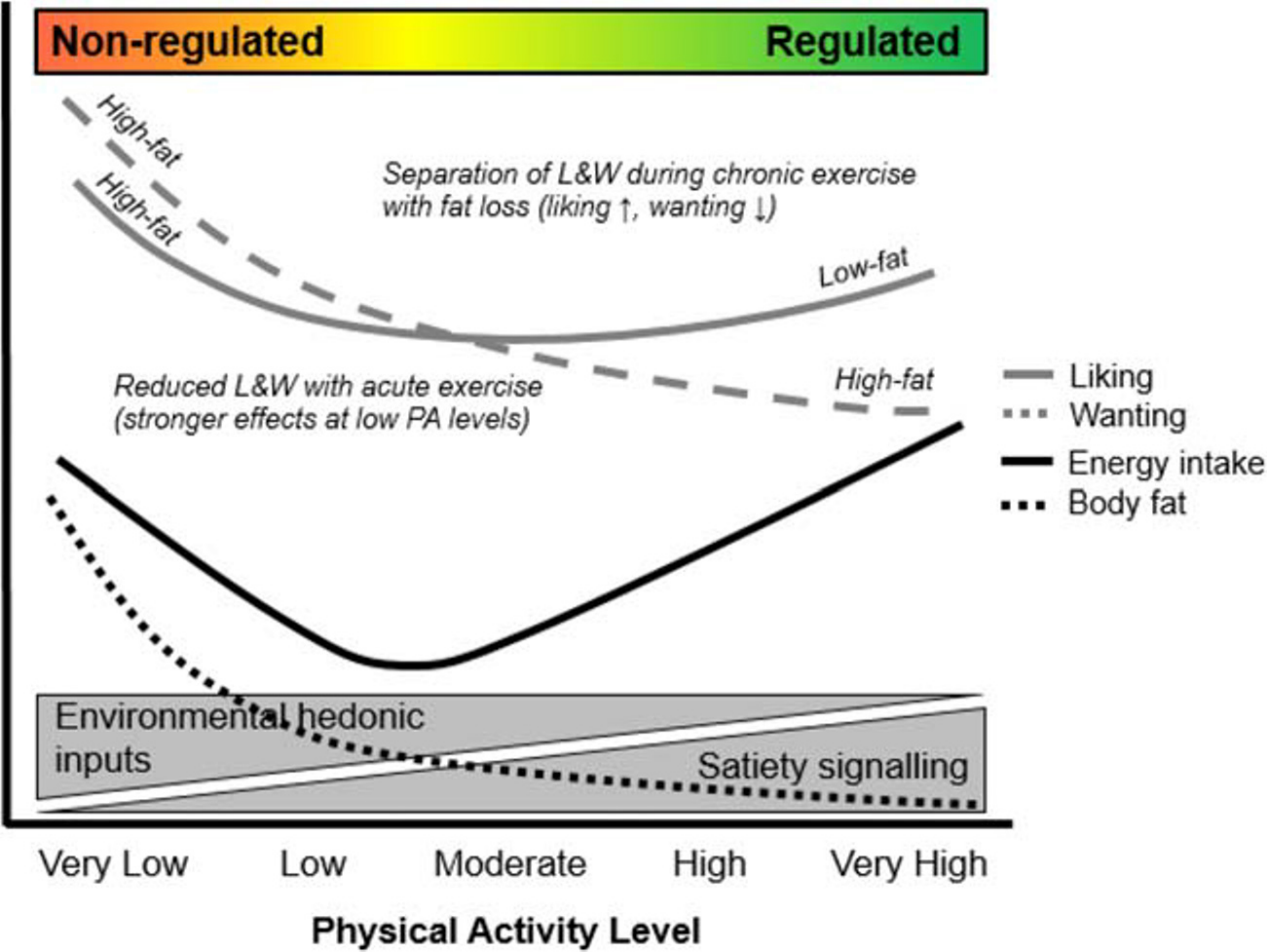
Conceptual model of the impact of habitual physical activity and exercise on appetite control. The model builds upon the relationship between physical activity level, energy intake, and body fat recently demonstrated by Beaulieu et al. [19••]. Higher levels of physical activity are associated with enhanced satiety signaling—within a “regulated zone” of appetite control—resulting in a better matching between energy intake and energy expenditure. Lower levels of physical activity are associated with higher body fat, weaker satiety signaling, and greater responsiveness to hedonic inputs from the food environment—within a “non-regulated zone” of appetite control—allowing for overconsumption to occur and body fat to increase further. This review adds to this model by proposing effects of physical activity on liking and wanting as processes of food reward. Specifically, lower levels of physical activity are associated with greater liking and wanting for high-fat/high-energy food, with wanting as the stronger driver of excess energy intake. Acute exercise leads to a reduction in liking and wanting, especially in inactive individuals. As habitual levels of physical activity increase (including during chronic exercise interventions), there is a small increase in liking and decrease in wanting that accompany weight loss and improvement in appetite control. Finally, higher levels of habitual physical activity (e.g., regular bouts of moderate-to-vigorous physical activity) are associated with greater liking for low-fat/low-energy food and lower wanting for high-fat food

One of the perceived barriers for engaging in exercise is its potential to promote hedonic eating. Food reward plays an important role in weight management through its intervening status between the nutrient requirements of the body and hedonic inputs from the food environment that promote food intake. This review brings together current evidence from observational, acute, and chronic exercise training studies to inform public debate on the impact of physical activity on food reward. A conceptual model, building on previous theory [19••] is shown in Fig. 2. Observational studies show that performance of moderate-to-vigorous physical activity is associated with lower liking and wanting for high-fat or high-energy food, and higher liking for low-fat/low-energy food. These findings may reflect improved appetite control and are supported by evidence from chronic exercise training interventions. Where exercise training leads to successful weight loss, it appears to be accompanied by a dissociation between liking and wanting evidenced by a reduction in wanting for high-energy food but increase in liking for low-energy food. Acute bouts of exercise tend to only impact behavioral indices of food reward in less active individuals or those with poor appetite control, where it tends to result in reduced food reward. These findings are corroborated by observational studies that demonstrate greater liking and especially wanting for high-energy foods (and greater susceptibility to food cravings) in inactive individuals. Food reward does not counteract the benefit of physical activity for obesity management. Rather, exercise appears to accompany positive changes in food preferences in line with improvements in appetite control.
